# Network collaboration of organisations for homeless individuals in the Montreal region

**DOI:** 10.5334/ijic.1138

**Published:** 2014-02-03

**Authors:** Marie-Josée Fleury, Guy Grenier, Alain Lesage, Nan Ma, André Ngamini Ngui

**Affiliations:** Department of Psychiatry, McGill University and Researcher, Douglas Hospital Research Centre, Montreal, Canada; Douglas Hospital Research Centre, Montreal, Canada; Department of Psychiatry, University of Montreal and Researcher, Fernand-Seguin Research Centre, L-H Lafontaine Hospital, Montréal, Canada; Fernand-Seguin Research Centre, L-H Lafontaine Hospital, Montreal, Canada; Douglas Hospital Research Centre, Montreal, Canada

**Keywords:** homelessness, network, interorganisational collaboration, determinants

## Abstract

**Introduction:**

We know little about the intensity and determinants of interorganisational collaboration within the homeless network. This study describes the characteristics and relationships (along with the variables predicting their degree of interorganisational collaboration) of 68 organisations of such a network in Montreal (Quebec, Canada).

**Theory and methods:**

Data were collected primarily through a self-administered questionnaire. Descriptive analyses were conducted followed by social network and multivariate analyses.

**Results:**

The Montreal homeless network has a high density (50.5%) and a decentralised structure and maintains a mostly informal collaboration with the public and cross-sectorial sectors. The network density showed more frequent contacts among four types of organisations which could point to the existence of cliques. Four variables predicted interorganisational collaboration: organisation type, number of services offered, volume of referrals and satisfaction with the relationships with public organisations.

**Conclusions and discussion:**

The Montreal homeless network seems adequate to address non-complex homelessness problems. Considering, however, that most homeless individuals present chronic and complex profiles, it appears necessary to have a more formal and better integrated network of homeless organisations, particularly in the health and social service sectors, in order to improve services.

## Introduction

Homelessness is an increasingly widespread social phenomenon in major urban centres. Montreal has one of the highest poverty rates in Canada [1] and is seriously affected by homelessness. In 2005, the number of homeless individuals in Canada was estimated at 150,000 [2], including some 30,000 (20%) in the Montreal area [3]. Homeless individuals often face co-occurring health and/or social problems and require, therefore, many services from organisations to meet needs such as food, housing, clothing, financial assistance, medical care and treatment for mental or addiction disorders. Since there is no single organisation that can deal with all these problems, homeless individuals often fall through the cracks. Hence, interorganisational collaboration is essential to meet the needs of this vulnerable population [4].

It is difficult to delimitate Montreal homeless organisations. In the Province of Quebec, homelessness is not one of the health care programmes of the ministry of health and social services. With the exception of men's shelters, few public or non-profit organisations serve homeless individuals exclusively. Homeless persons with mental, physical or addiction disorders use the same resources as individuals who may or may not be at risk of becoming homeless. However, studying this network could be instructive because it comprises various organisations presenting distinct characteristics and serving the most vulnerable individuals with concurrent social and health problems.

As for other vulnerable populations (e.g. the frail elderly, individuals with serious mental disorders or chronic diseases) in Western countries, most of the homeless persons’ needs (e.g. food, money, accommodation, social support) are covered mainly by non-profit organisations while public institutions address health needs [5]. This fragmentation of services can seriously impair the continuity of care [6]. Moreover, the Montreal homeless organisations, especially non-profit organisations, have limited resources [7]. With an integrated network of organisations, the issue of service fragmentation would fade [8]. Since the 1990s, cooperation between organisations increased as integrated networks for chronic and complex problems were set up. This was one of the aims of health care reforms in Quebec [9, 10], Canada [11] and many other countries [12]. Integrated networks involve autonomous organisations that collaborate interdependently [4]. According to the literature, organisations can respond more efficiently to the needs of the public if they work together instead of competing or acting independently [13, 14]. Some of the presumed benefits of integration are higher quality of care, wider access to services, more client satisfaction and cost reductions [14, 15]. Networks appear more flexible and adaptable, but also more fragile, than hierarchical structures [4].

In a network, interorganisational interactions can take several forms including informal and contractual relationships and mergers [16, 17]. In his framework, Whetten [18] identified three types of interorganisational relationships: mutual adjustment, alliance and corporate structure [19]. In mutual adjustment, there are no coordination mechanisms between organisations and their relationship (e.g. sharing of information on clientele) is essentially informal. In alliance, coordination is more formalised and derives from negotiation between organisations. Finally, the corporate structure is the most formalised interorganisational relationship and includes a hierarchical structure of governance and a system of sanctions [19]. For his part, Konrad describes integration or interorganisational relationships according to a five-level continuum: information sharing and communication, cooperation and coordination, collaboration, consolidation and integration [20]. Information sharing and communication as well as cooperation and coordination are the least intense levels of integration and involve mostly informal interorganisational relationships. At the consolidation and integration levels, the relationships are highly formalised and structured. Finally, at the collaboration level, formal and informal relationships coexist [20]; organisations remain autonomous but pursue mutual goals [21, 22].

The nature and the intensity of interorganisational collaboration among Montreal homeless organisations are however unknown. According to the literature, there would be fragmentation and lack of coordination in the delivery of services from homelessness [14]. Learning more about interorganisational collaboration could facilitate planning and evaluation [13]. Social network analysis offers an effective method for investigating such relationships [23]. It allows an analysis of the number, type and extent of relationships between organisations within a network [5]. It also measures the degree of contact between individuals or organisations and generates valuable information regarding, for instance, the existence of cliques within the network and the organisations occupying the centre of the network [24]. In the health and social service sectors, this method proved useful in analysing collaboration between organisations such as human immunodeficiency virus/acquired immunodeficiency syndrome service agencies [25], women's groups [26], chronic disease services [8], tobacco control networks [23], primary care practices [27] and health advocacy coalitions [28]. However, there has not yet been a specific analysis of social network of collaboration among organisations serving homeless individuals.

Determinants of interorganisational collaboration, particularly between non-profit and public organisations, have been the object of various studies [29–32]. Interorganisational collaboration can be influenced by factors like ideology [33], age of the organisations [30, 31], history of collaboration [34], volume of referrals [35] or profile of the clientele [36]. Non-profit organisations that establish formal relationships with public organisations generally receive government funding [30, 36]. Most authors believe that organisations with fewer resources tend to collaborate more often with public organisations [32, 36, 37], but other researchers have concluded that it is organisations with a larger budget that are more likely to cooperate with the public sector [31]. Furthermore, to our knowledge, determinants of collaboration among organisations serving the homeless or persons at risk of becoming homeless have not been the subject of a specific study.

As part of a broader research and demonstration project (The At-Home/Chez Soi Project) on mental health and homelessness [38], and based on a survey of 68 organisations (64 non-profits and four public) serving homeless individuals or persons at risk of becoming homeless, this study aimed to describe the characteristics of organisations, to examine interorganisational collaboration between Montreal homeless organisations using a social network analysis, and to identify variables associated with interorganisational collaboration.

## Method

### Sample

Montreal is one of the five settings chosen by the Mental Health Commission of Canada to perform a research and demonstration project on mental health and homelessness [38]. Montreal is the second largest city in Canada, and Quebec's largest, with a population of 1,800,000 or 25% of Quebec residents. It covers 12 health local services networks.

Our cross-sectional study was conducted from October 2010 to November 2011 among 152 non-profit organisations and four public facilities (three health and social service centres [HSSCs] and one specialised addiction centre) that serve homeless individuals or persons at risk of becoming homeless in the Montreal region. These organisations (Montreal homeless organisations) were selected from a pre-established list of organisations funded by the Quebec government, and from a directory of non-profit homeless organisations. To be included in the study, organisations had to meet the following criteria: being recognised and funded by the Quebec government, offering services to the homeless or to people at risk of homelessness, being situated in the Montreal region and having submitted an annual activity report to the regional health agency.

### Measurement tools

Data collection was based on the 2008–2009 activity reports from organisations and a self-administered questionnaire adapted from previous research [39] and validated by a steering committee of 11 stakeholders with expertise in homelessness. From the activity reports provided by the Montreal Regional Agency, information such as annual budget, number of services provided and chief operations were extracted. Supplementary information about budgets was extracted from the registered charity information return of the Canada Revenue Agency [40]. The questionnaire, which required about 45 minutes to complete, consisted of categorical or continuous items with either 3- or 5-point Likert-type scale questions (0 = never, 1 = occasionally, 2 = often; 1 = highly inadequate, 2 = inadequate, 3 = correct, 4 = adequate, 5 = highly adequate). Items were grouped under eight headings: (1) information on the respondent; (2) territories covered; (3) organisational characteristics; (4) user profiles during the last 12 months; (5) internal functioning; (6) financing; (7) interorganisational relations and satisfaction; and (8) accessibility to health and social services. Since the scales between items were different, the last two headings were transferred on a scale of 0 to 100 for data comparison. The questionnaire was pre-tested on eight organisations and delivered by mail. There was an average of four follow-ups (minimum = 0; maximum = 7). To ensure confidentiality of data, each organisation was assigned a code. Five relevant ethics boards approved the study protocol.

#### Definition of variables and statistical analyses

Descriptive analyses were conducted first, followed by social network and multivariate analyses. The intensity of collaboration between Montreal homeless organisations and the public sector as well as collaboration within the Montreal homeless organisation network were explored using UCINET software. Dependent variable was interorganisational collaboration. It was estimated based on the following question: ‘Among the organisations listed (or others that you can add), which are the ones with which you have significant and recurrent collaboration?’ Collaboration included participation in councils and meetings, referrals provided and received, signed agreements and informal collaboration. The total score of interorganisational collaboration could vary from 0 to a maximum of 336. The question above was subdivided to consider collaboration within the voluntary sector and between the latter and the public sector. Comparison involved eight types of Montreal homeless organisations based on the following Quebec ministry of health classifications: (1) basic subsistence needs (e.g. food banks, clothing stores); (2) mental health (e.g. crisis centres, housing, day centres); (3) temporary housing for women who are victimised or in difficulty (e.g. women's shelters, resources for women victims of spousal violence); (4) human immunodeficiency virus or acquired immunodeficiency syndrome services (e.g. rights defense for sex workers, housing for individuals with acquired immunodeficiency syndrome); (5) youth resources (e.g. shelters for young people, youth hostels); (6) addiction services (e.g. specialised addiction centre, day centres for addiction); (7) men's shelters; and 8) HSSCs (e.g. outreach teams, psychosocial services).

UCINET is a social network analysis software that illustrates the relationships among network members both by means of graphics and statistical analysis [41]. In a network graph, each organisation represents a node, interconnected with other nodes via a line, called a tie. A tie shows the presence, direction or strength of the relationship. The graphic representation of the relationships among members of a network allows visualising first whether there are one or more networks and, second, the symmetry of the relationships between members. All Montreal homeless organisations were assigned a number to ensure confidentiality. Four statistics were considered: density, centrality, in- and out-degree centralities. Density is the number of ties that are present as a proportion of total possible ties. A higher density value reflects more ties. Density scores ranged between 0 and 100%. Centrality refers to the number of ties or links one organisation has with other organisations in the network in relation to other organisations’ number of ties or links. The organisation with the most ties has the highest degree of centrality and also occupies the most central part of the graph. In-degree centrality is the number of ties credited to a given responding organisation by other respondents while out-degree centrality refers to the ties that a respondent organisation reported having with other organisations. Centrality scores for this measurement count as a percentage that ranges between 0 and 100%.

Interpretation of the graphs and various centrality scores is not standardised, even though terms have been suggested [42, 43]. A perfect symmetrical and unique web would reflect a fully integrated network. In a paper describing primary care organisations, Scott et al. [27] stressed that equilibrium between in- and out-degree centralities would reflect a more collaborative, less hierarchical system. Dekker [44] reported that centrality scores reflect the status of an organisation in a network: the lower and higher scores depend on the percentage of ties in a given network. The centrality score for an entire network expresses the degree of collaboration and integration of that network.

**Figure 1.**
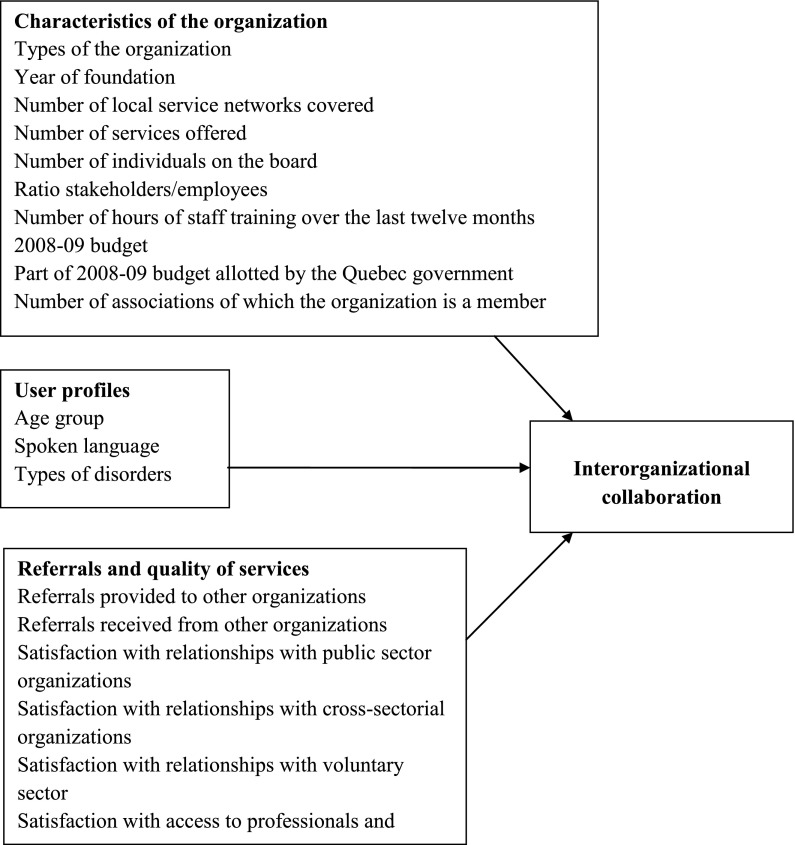
Analytic model of determinants of interorganizational collaboration.

Multivariate analyses were also conducted, following the analytical framework displayed in Figure 1, to identify determinants of interorganisational collaboration within the homelessness network. Independent variables were organised in accordance with the three categories of the framework: characteristics of the organisation, user profiles and referrals, as well as quality of service. Given their heterogeneity as to size and mission, organisations were asked to report on their frequency of collaboration with other resources using a Likert scale ranging from 0 to 2 (never; occasionally; often). Responses were aggregated in a continuous variable with potential values ranging from 0 to 225.

Statistical analyses were tailored to the dependent variable and encompassed univariate, bivariate and multivariate analyses. Since the dependent variable was continuous, normality assumption was first tested. Univariate analyses comprised frequency distribution for categorical variables and mean and standard deviation values for continuous variables. Appropriate tests (Pearson correlation or chi-square test) [45] were used in bivariate analyses to assess variables showing a significant link with the outcome. At this stage, we considered the level of 0.07 or less to be significantly associated with the independent variable. Before the final multivariate analyses, each block of the conceptual framework was assessed independently starting from block one to block three and only significant variables were retained for the second round of analyses. In the second round, significant variables in block one and block two were analysed together, and significant variables were aggregated with significant variables of block three. They were then introduced into a multivariate linear regression using a backward solution to avoid type II error [46]. Participating organisations were also divided into two groups based on the mean of significant collaboration of the entire sample. Those who scored below the mean were labelled ‘under’ and the rest were labelled ‘above.’ Analyses were conducted using SAS 9.2^®^ for Windows [47].

## Results

### Description of the sample

Forty-four organisations were excluded from the total sample of 156 because they did not meet the selection criteria listed in the methods or did not offer direct services to the population or were a subdivision of an organisation already listed. Of the 112 remaining organisations (108 non-profits and four public), 68 including the four public facilities returned the questionnaire, for a response rate of 61%. The 68 respondents were divided according to the eight organisational groups as follows: (1) basic subsistence needs (*n* = 15); (2) mental health (*n* = 13); (3) temporary housing for women who are victimised or in difficulty (*n* = 9); (4) human immunodeficiency virus or acquired immunodeficiency syndrome services (*n* = 9); (5) youth resources (*n* = 7); (6) addiction services (*n* = 6); (7) men's shelters (*n* = 6); and (8) HSSCs (*n* = 3).

HSSCs have the highest overall budget, followed by men's shelters, temporary housing for women who are victimised or in difficulty, human immunodeficiency virus/acquired immunodeficiency syndrome service organisations, mental health organisations, youth resources, addiction service organisations and finally basic subsistence needs organisations. The median of organisations’ global budgets was CAN$570,821.50. However, 73% of basic subsistence needs organisations had a global budget of less than CAN$300,000. The Quebec ministry of health and social services provided on average 33% of organisations’ budgets for the voluntary sector (versus 100% for the public sector). On average, the proportion of organisations’ funds provided by the ministry of health and social services was higher for mental health (53%), youth (47%), temporary housing for women who are victimised or in difficulty (44%) and human immunodeficiency virus/acquired immunodeficiency syndrome (39%). However, the ratio was only 12% for men's shelters and 8% for addiction services and basic subsistence needs, respectively. Furthermore, federal and municipal governments provided, respectively, 6% and 5% of organisations’ global budgets. Only 31% of organisations believed that their level of funding was adequate or highly adequate – mainly temporary housing for women who are victimised or in difficulty (71%). The most unsatisfied organisations were men's shelters, addiction service organisations and HSSCs, none of which felt that their level of funding was sufficient.

Organisations covered an average of 10 local health networks. However, most basic subsistence needs organisations (77%), youth organisations (71%), men's shelters (67%), HSSCs (67%) and a sizeable proportion of mental health organisations (42%) served only one or two local health networks. Montreal homeless organisations offered on average 5.8 services ± 2.6 (e.g. food, housing, follow-up in the community). Men's shelters offered the most services on average (8.5 ± 2.7). Inversely, organisations offering youth services (4.4 ± 1.0) and basic subsistence needs assistance (4.6 ± 2.4) provided the fewest services. Most organisations (59%) were members of the *Réseau d'aide aux personnes seules et itinérantes de Montréal* (RAPSIM: Montreal's assistance network for single, homeless persons) – a group of local organisations acting on behalf of the homeless. While most organisations (78%) were created after 1981, almost all men's shelters predate that year (5/6; 83%). On average, 42% of clients were homeless and 40% were at risk of becoming homeless. As Table 1 indicates, individuals aged between 25 and 50 were the highest users of services offered by these organisations, and clients faced a number of health or social problems including gambling (10%), alcohol or drug abuse (36%), common mental disorders (e.g. anxiety) or legal difficulties (33%), severe mental disorders (e.g. schizophrenia) (24%) and spousal or family violence (18%). The organisations’ clientele was predominantly male (59%) and French-speaking (74%).

Table 2 displays the mean and standard deviations of referrals provided and received by different organisations. On one hand, youth resources organisations were those that most often provided (44.71 ± 8.12) and received referrals (33.57 ± 8.90), followed by men's shelters (provided: 41.5 ± 11.26; received: 32.33 ± 8.26). Mental health organisations provided and received fewer referrals than others (provided: 20.07 ± 9.88; received: 20.46 ± 10.33). On the other hand, public organisations received the most referrals from both youth resources (12.86 ± 2.41) and temporary housing for women who are victimised or in difficulty (12.11 ± 3.76). Meanwhile, non-profit organisations received most of their referrals from youth resources (24.14 ± 4.09).

Regarding satisfaction with interorganisational relationships, on a scale of 0 to 100, the average score was 61 (75 within the voluntary sector, 67 within cross-sectorial organisations [i.e. housing, employment, justice, and education] and 58 within the public sector). On the same scale, adequacy of access to comprehensive services and professionals for the homeless received an average score of 35. Access to general practitioners and to psychiatrists was deemed highly inadequate, respectively, by 91% and 78% of organisations. As regards access to various types of services, the following were considered highly inadequate or inadequate: private housing (by 81% of the organisations), social housing (76%) and assisted living (58%). Overall satisfaction regarding access to services was higher among HSSCs (89.7% satisfactory to highly satisfactory), youth organisations, (84%), men's shelters (79%), temporary housing for women who are victimised or in difficulty (78%) and mental health organisations (71%). Organisations offering basic subsistence needs assistance (60%), addiction services (58%) and human immunodeficiency virus/acquired immunodeficiency syndrome services (55%) were the least satisfied.

### Network dynamics – social network analysis

**Figure 2.**
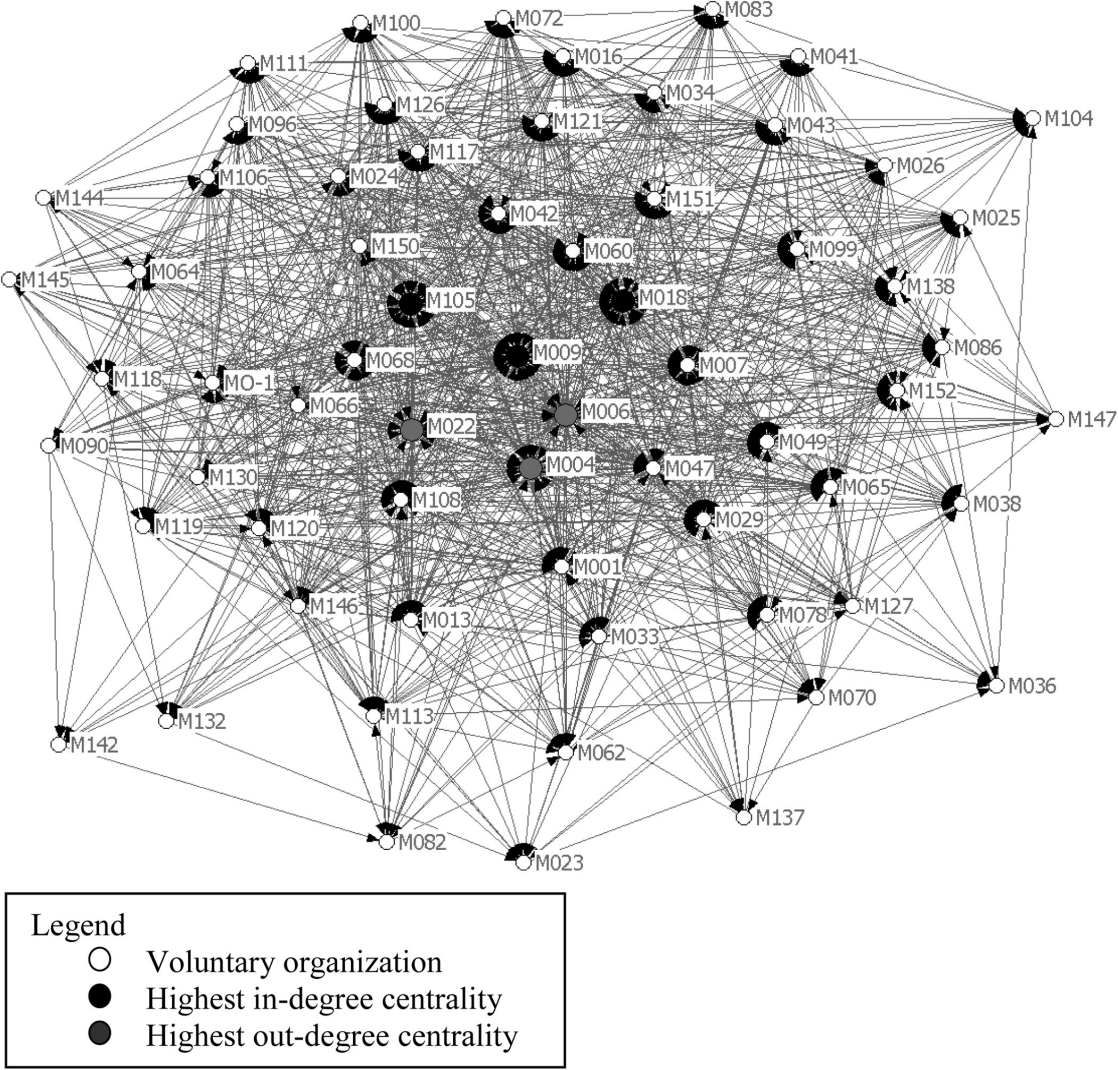
Network diagram: interorganizational collaboration between Montreal organizations active in the homelessness sector.

Figure 2 illustrates the collaborative patterns of the Montreal homeless organisation network. All organisations represented in the graph had direct or indirect ties in this network. The overall in-degree centrality score was 27.5% versus 65.1% for out-degree centrality and 50.5% for network density. To determine the most and least central organisations in this network, the three organisations with the highest in-degree or out-degree centrality scores and the top and bottom 20th percentile values were used as cut-off points. The three organisations with the highest in-degree centrality were organisations M009, M018 and M105 (67%, 64% and 60% of in-degree centrality, respectively) meaning they were contacted most often by other organisations. These three organisations provided services to people with mental health problems, addictions and youth, respectively. The three organisations with the highest out-degree scores were identified as M006, M004 and M022 (95%, 85%, and 84% of out-degree centrality, respectively). They had frequent contacts with other organisations and their services focused on basic subsistence needs, human immunodeficiency virus/acquired immunodeficiency syndrome and men's shelters. The 18 organisations with the lowest in-degree centrality represented almost every type of volunteer group but especially those involved in basic subsistence needs and mental health. The 22 organisations with the lowest out-degree centrality dealt mainly with basic subsistence needs and mental health.

**Figure 3.**
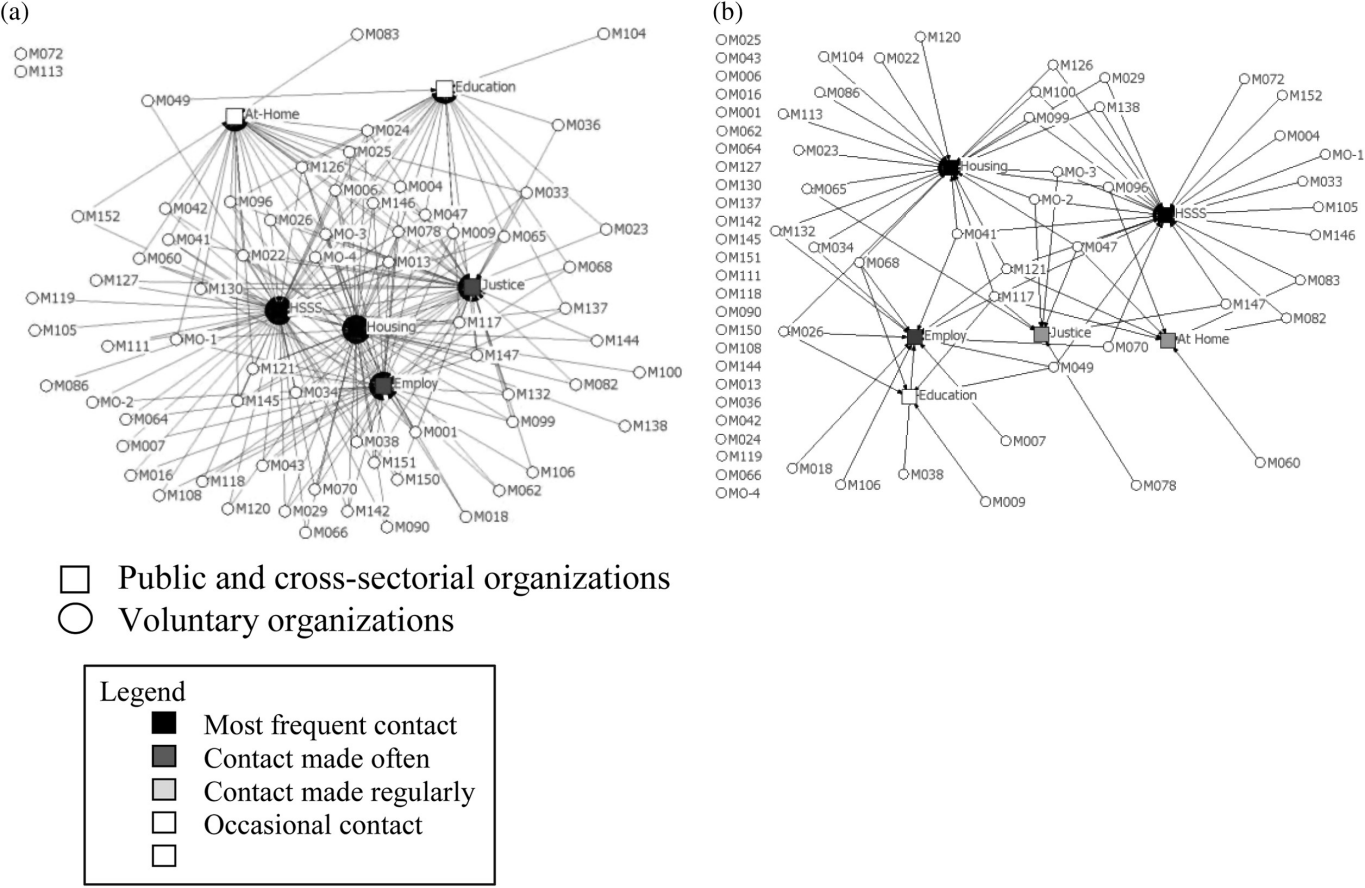
Network diagram: (a) informal and (b) formal collaboration between the voluntary sector and public and cross-sectorial organizations.

Figures 3a and b compare patterns of informal and formal collaboration between Montreal homeless organisations and the public sector. In terms of network density, the rate of informal collaboration was 54.2%, compared to 19.4% for formal collaboration. Figure 3a shows the centrality of the public organisations within the voluntary sector's network. The most frequent contacts were initiated by health and social service organisations and the housing sector and to a lesser extent by the employment and justice sectors (Table 3). The education sector and the At-Home/Chez Soi project were both located at the network's periphery. Two non-profit organisations providing mental health services had no contact with the public sector.

Figure 3b (formal collaboration) shows fewer relationships compared with Figure 3a (informal collaboration). Forty-two of the 64 non-profit organisations (65.6%) signed at least one formal agreement while eight signed more than three protocols with different public organisations. All the addiction service organisations (100%) and most mental health organisations (84%) and men's shelters (83%) signed at least one protocol. Inversely, most human immunodeficiency virus/acquired immunodeficiency syndrome services (89%) and basic subsistence needs organisations (53%) had no formal agreement. The public and cross-sectorial organisations with the highest formal collaboration were in the health and social service sector, with a network centrality of 35.3% followed by housing (33.8%) and employment (19%), the At Home/Chez Soi Project and the justice sector (about 10%). The lowest score occurred within the education sector (7.45%).

**Figure 4.**
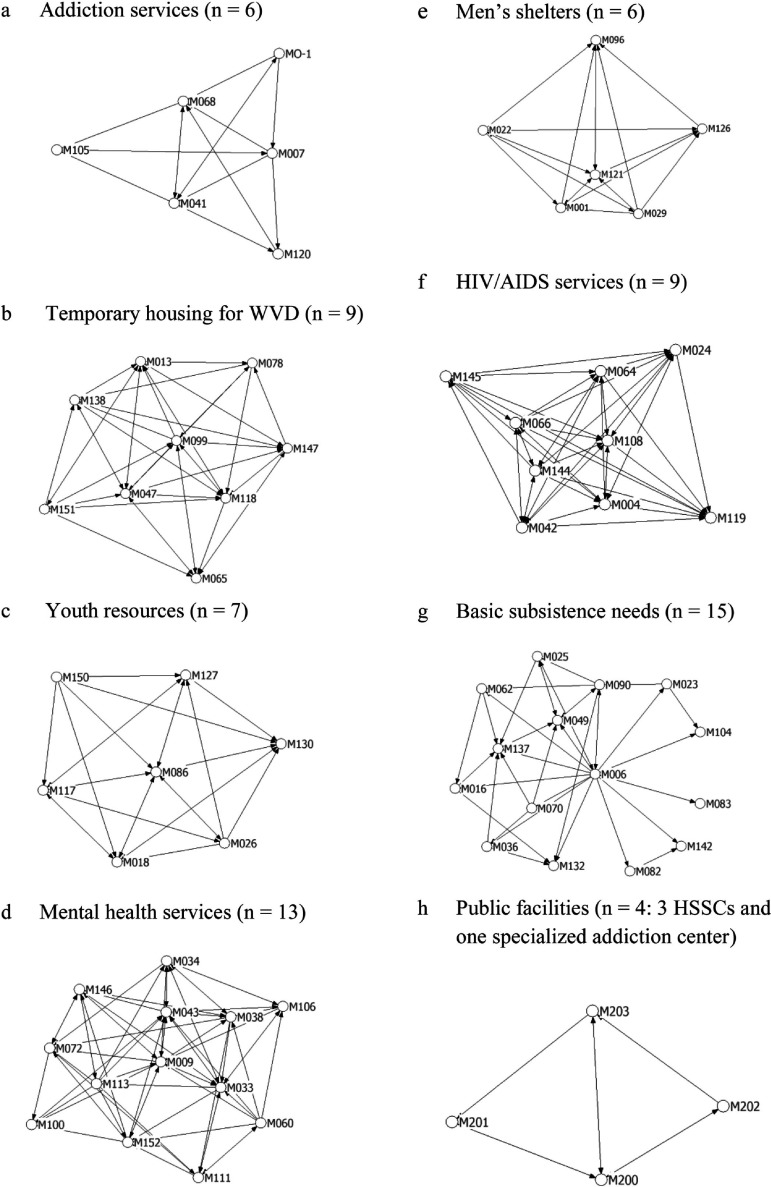
Graphical display of interorganizational contacts of eight organization types. (a) Addiction services (*n* = 6), (b) temporary housing for women who are victimized or in difficulty (*n* = 9), (c) youth resources (*n* = 7), (d) mental health services (*n* = 13), (e) men's shelters (*n* = 6), (f) HIV/AIDS services (*n* = 9), (g) basic subsistence needs (*n* = 15), (h) public facilities (*n* = 4: 3 HSSCs and one specialized addiction centre).

Figure 4 shows the network structure and cohesion for each type of Montreal homeless organisation. The network density showed frequent contacts among four types of organisations: human immunodeficiency virus/acquired immunodeficiency syndrome (76.5%), public facilities (including the three HSSCs and the specialised addiction centre, 68.9%), temporary housing for women who are victimised or in difficulty (64.2%) and men's shelters (61.1%). Youth resources (50.0%), addiction services (44.4%) and mental health organisations (37.3%) showed a more moderate contact rate while organisations providing basic subsistence needs assistance showed the lowest contact rate (16.9%).

### Bivariate and multivariate analysis

Bivariate analyses yielded eight independent variables significantly associated with the intensity of collaboration among organisations. Two relate to the type of organisation (*p* = 0.001) and number of services offered (*p* < 0.001); two relate to user profile: percentage of clients having legal problems (*p* = 0.03) and percentage of clients with gambling problems (*p* = 0.03). The four other variables related to referrals and quality of services: referrals received from other organisations (*p* < 0.001), referrals provided to other organisations (*p* = 0.004), satisfaction with access to local professionals (e.g. general practitioners, psychiatrists) (*p* = 0.01) and satisfaction with relationships with the public sector (*p* < 0.001).

As shown in Table 4, 93.3% of the basic subsistence needs organisations performed under the global mean of collaboration versus none of the HSSCs. Organisations that performed above the mean offered about 6.4 services; a mean average of 37.7% of their clients were homeless people with legal problems. These organisations would also make more referrals to other groups. Their mean of referral was 35.1 with a standard deviate of 15.6. The final multivariate model (Table 4) contained three variables positively associated with interorganisational collaboration and accounted for 38.5% of the total variance. After controlling for variables associated with interorganisational collaboration, for each additional level of services offered, interorganisational collaboration increased by 9.90 (*p* < 0.05). Higher satisfaction with relationships with the public sector increased collaboration (*β* = 2.41, *p* < 0.05). Finally, organisations that received more referrals from others tended to achieve the highest degree of collaboration (*β* = 1.10, *p* < 0.05).

## Discussion

The purpose of this study was to describe the characteristics of organisations, examine interorganisational collaboration between Montreal homeless organisations using a social network analysis and identify variables predicting interorganisational collaboration. It draws its results from a survey of 68 organisations (64 non-profits and 4 public) serving homeless individuals or persons at risk of becoming homeless.

The response rate (61%) was satisfactory, compared with most studies concerning the non-profit and voluntary sectors [35, 48, 49]. Usually, response rates for mail surveys from organisations are lower than those from individuals [50].

### Characteristics of Montreal homeless organisations

One of the main characteristics of Montreal homeless organisations was the number of local service networks they served (mean = 10). Usually, HSSCs and most non-profit organisations have a local or a semi-regional mandate. The areas that most Montreal homeless organisations covered were in the downtown area – where the homeless converge – but also at the outskirts of the city, thus indicating the existence of several poor neighbourhoods around Montreal.

Another aspect of Montreal homeless organisations was that they received little funding (33% of their general budget) from the ministry of health and social services compared to some health programmes. For example, a recent study among Quebec mental health non-profit organisations revealed that 64% of their budget came from the ministry of health and social services [39]. Hence, it appears that some organisations, especially basic subsistence needs organisations, addiction services and men's shelters, depend mostly on donations from individuals and private foundations rather than government funding. These Montreal homeless organisations did receive funding from federal and municipal governments, but it was usually below the level of provincial funding.

The proportion of Montreal homeless organisations clients with mental health problems, addiction or social problems is quite similar to that found in the international literature [51]. The presence of severe concurrent problems explains – and probably justifies – the need for these organisations to collaborate. Concerning referrals, it seems remarkable at first that mental health organisations provide and receive fewer referrals than other types of organisations. A possible explanation would be that some organisations only provide referrals while others only receive them. The graph in Figure 4d seems to confirm this hypothesis: the two organisations offering follow-up in the community (M060 and M113) were in contact with several other organisations, but were not often contacted. Inversely, most organisations offering long-term housing (*n* = 6) were contacted by several mental health organisations, but there was no reciprocity. Non-profit organisations offering long-term housing usually accommodate and support a relatively small number of clients, thus meeting almost all their needs. Another possible explanation would be that most of those non-profit organisations were initially created to provide an alternative to traditional psychiatric hospitals [33] and are, therefore, reluctant to refer clients to public mental health institutions.

### Montreal homeless organisation network dynamics

Montreal homeless organisations are characterised by their network's density (50.5%), which was higher or equivalent to results (usually in the 30%) from other studies using a social network analysis [23, 25–27]. The high density of the Montreal homeless organisation's network was noteworthy since it comprises public and non-profit organisations involved in a variety of programmes (mental health, addiction, human immunodeficiency virus/acquired immunodeficiency syndrome, etc.). The multiple connections between Montreal homeless organisations confirmed its status as a structured network as defined by Phillips [26]. Moreover, the presence of many two-way arrows indicated that the structure of the Montreal homeless organisation network was not hierarchical [27]. The fact that most organisations were members of the Réseau d'aide aux personnes seules et itinérantes de Montréal explains this high density. This group plays a liaison role between the Montreal homeless organisations [1]. A high density can have positive, but also negative aspects [43, 52]. It might indicate the existence of shared values and beliefs among members of the Montreal homeless organisation network, which facilitate information sharing and decision-making [43]. It is possible that high density of the Montreal homeless organisation network would be beneficial for homeless individuals, since it might reduce the risk of falling through the cracks, except when they refuse services. However, connections with external resources can also be restricted in a high-density network [43]. Within the Montreal homeless organisation network, there is little collaboration and few formal relationships with the public health sector, meaning perhaps that many homeless persons with chronic and complex profiles have no access to health and social services. Studies that focused on the link between collaboration and user outcomes have arrived at conflicting results. Some studies revealed that collaboration between organisations or professionals would increase patient satisfaction [52] and reduce death rates [53], length of hospital stays and hospitalisation costs [54, 55]. Other studies have indicated, however, that other factors such as stability or level of funding can affect the positive association between integration and outcome [7, 56]. Finally, according to other studies, the impact of integration on user services and outcomes is limited [57, 58]. Moreover, performance of a network can be compromised if its density is too high. For example, in a study on a physician collaboration network, Uddin et al. [52] found that density increased hospitalisation costs and readmission rates. As the number of organisations in a network increases, it becomes more difficult for them to work harmoniously [14].

The Montreal homeless network was highly decentralised, ‘with a polycephalous leadership’ [13, p. 769]. This particularity can be explained by the historical context. In 1988, the city of Montreal set up an organisation (*Dernier Recours*) to coordinate interventions in the field of the homelessness [59]. This experience failed and was abandoned three years later. Using Whetten's framework on the types of network coordination [18], we can conclude that the current structure of Montreal homeless organisation network is of the ‘mutual-adjustment’ type. Under this last form of coordination, the exchanges between Montreal homeless organisations are generally voluntary and imply no formal means of coordination [19].

The most central organisations with the highest in-degree or out-degree were not limited to a specific type of Montreal homeless organisation. The three organisations most frequently contacted by Montreal homeless organisations provided services, respectively, to people with mental health issues, addictions and youth. They have a point in common in that they offer transitional or long-term housing. It was more difficult to identify common characteristics of the three organisations with the highest out-degree scores. However, all three organisations reported that most of their clients were homeless (80%, 66% and 90%, respectively) and had legal problems (60%, 89% and 75%, respectively). Moreover, according to two of these organisations, the percentage of clients with an addiction disorder was 86% and 100%, respectively. The incidence of multiple health and social problems among their clientele may explain their strong need to contact various organisations.

Collaboration between the voluntary sector and the public or cross-sectorial sectors is usually informal [31]. Interorganisational relationships between Montreal homeless organisations and their partners were no exception. Although the vast majority of Montreal homeless organisations reported informal relationships with public organisations, there are relatively few formal agreements. Several non-profit organisations were reluctant to formalise their relationships with public organisations [60], mainly from fear of losing their autonomy [29, 61]. This is the case namely of organisations offering few professional services [62]. However, government funding can be an incentive to the elaboration of formal relationships between non-profit and public organisations [30, 36]. Interestingly, public organisations that had the highest level of formal collaboration (health and social service organisations and housing sector) also had the highest level of informal collaboration. For several organisations, informal collaboration may be the first step towards signing a formal agreement.

The specific networks for the eight types of Montreal homeless organisations had varied patterns. Networks comprising the four types of organisations with the highest overall budget (human immunodeficiency virus/acquired immunodeficiency syndrome services, public facilities, temporary housings for women who are victimised or in difficulty and men's shelters) had a stronger density than the general Montreal homeless organisation network. At first view, those results seems to confirm the hypothesis that organisations with the highest budget are more likely to cooperate [31]. However, such high density could also indicate the existence of cliques, [7], thus indicating a fragmented network. Cliques may be the result of the maturity of organisations, their history of collaboration or their sharing of common values [30–32, 63]. The number of human immunodeficiency virus/acquired immunodeficiency syndrome service organisations in Quebec grew considerably, along with their funding, when they received the mandate in the 1980s to provide and manage housing for persons with acquired immunodeficiency syndrome [64]. As indicated previously, these organisations were typically unsatisfied of their relationships with public organisations and their collaboration with them was usually informal. Since they were probably more radical than others, this could explain their conflicting relationships with the public sector [33]. The high density between public facilities is understandable since these organisations have common aims, values and practices [63]. The high density between organisations offering temporary housing for women who are victimised or in difficulty could stem from a sense a collective identity linked to feminist ideology [26]. According to Foster and Meinhard [30], women-led organisations are more likely to cooperate. Finally, men's shelters are older and have a long history of collaboration. Moreover, those organisations were all established by religious or philanthropic communities and, therefore, share common values [65].

As regards addiction service organisations and mental health organisations, the density was a little lower than the general density of the Montreal homeless organisation network. The moderate density within the addiction service network could be the consequence of the small operating budget available to these organisations. As indicated previously, the prevalence of unidirectional contacts between mental health organisations could explain the moderate density in the mental health network. Moreover, unlike other health programmes, the mental health network exists mainly on a local or semi-regional basis. Several organisations have similar missions (e.g. crisis centre, follow-up in the community) and intervene in specific local services networks. When a need arises, a day centre will contact the nearest crisis centre and not others.

Finally, the density among basic subsistence needs organisations was particularly low (16.9%). There were also few two-way arrows, which confirm the presence of a hierarchical structure. Most of these organisations (60%) serve only one local service network and offered a limited number of services (food, clothes, etc.) and they operate with the most limited budgets [26]. These two factors could explain this network's low density.

### Determinants of interorganisational collaboration between Montreal homeless organisations

Interorganisational collaboration was found to be influenced by two organisational characteristics: type of organisation and number of services offered. Concerning the type of organisations, it is logical that HSSCs would collaborate to a higher degree with their various partners. The mandate of HSSCs is to improve service integration between agencies serving a particular local service network. Furthermore, the multivariate analysis confirmed the results of the social network analysis showing that basic subsistence needs organisations collaborated much less than other organisations. A significant number of mental health or human immunodeficiency virus/acquired immunodeficiency syndrome service organisations also performed under the global mean of collaboration. As indicated previously, these were typically organisations that accommodated and supported a small clientele. Concerning the number of services offered, Foster and Meinhard [30] have reported that organisations offering numerous services are more attractive for prospective partners and are thus more likely to cooperate. The lack of resources is an incentive to cooperate, but a minimum of services is also necessary to support such collaboration [56]. The number of services is indirectly linked to the size of the budget, which explains why larger organisations were more likely to cooperate.

The connection between interorganisational collaboration and referrals received from other organisations makes sense. Referrals are the main type of interorganisational collaboration, and require the lowest degree of mutual dependency [66]. The involvement of organisations that provide referrals to or get referrals from other organisations is limited, and usually does not require a formal agreement [35]. Nevertheless, having multiple referral sources improves the legitimacy and viability of the organisation [60].

Finally, the link between interorganisational collaboration and satisfaction with relationships with the public sector is also understandable. As indicated previously, collaboration between non-profit and public organisations is largely informal and based primarily on mutual trust and quality of personal relationships [25]. Mutual trust can also be perceived as an outcome of interorganisational collaboration [67].

## Limitations

This study includes specific limitations that are worth noting. First, the number of clients for each Montreal homeless organisations and the exact percentage of those referred to the other organisations were unknown. Second, while we knew what the overall budget was for each Montreal homeless organisation, it was impossible to determine the amount dedicated specifically to homelessness. Third, while there were numerous interorganisational relationships among Montreal homeless organisations, further study is needed to see if a high-density network is more effective in meeting the needs of the homeless. Fourth, a qualitative data set could have helped our interpretation as it would have allowed a more detailed description of each type of interorganisational collaboration. Finally, with a longitudinal study, we could have more effectively isolated factors associated with interorganisational collaboration.

## Conclusion

This study is the first to our knowledge to analyse the social network of organisations serving homeless individuals or persons at risk of becoming homeless, and to identify factors associated with their interorganisational collaboration. Better understanding of interorganisational collaboration could facilitate planning of homelessness sector and improve services offered to this vulnerable clientele. Our results show that the Montreal homeless network has a high density which it comprises different non-profit and public organisations with distinct characteristics, and is highly decentralised. The density of a network could be a positive or a negative factor for homeless individuals. As interorganisational relationships with the public health sector are limited and rarely formalised, some homeless persons with chronic and complex profiles could end up having no access to health and social services. Moreover, some types of organisations are less likely to collaborate while others seem to develop cliques within the homelessness network. According to Whetten's framework [68], the Montreal homeless organisations network is of a ‘mutual-adjustment’ type. This model is usually appropriate for non-complex problems. However, considering that most homeless individuals present chronic and complex profiles, greater formalisation and better integration of the Montreal homeless organisations network, particularly between health and social service sectors, seems necessary to improve services. Several integrated strategies, such as liaison officers, centralised governing structure and interorganisational training [14, 68, 69], could also be introduced to increase the capacity of the various organisations to collaborate. According the literature, it is more effective to use multiples strategies than a single one [68].

## Figures and Tables

**Table 1.**
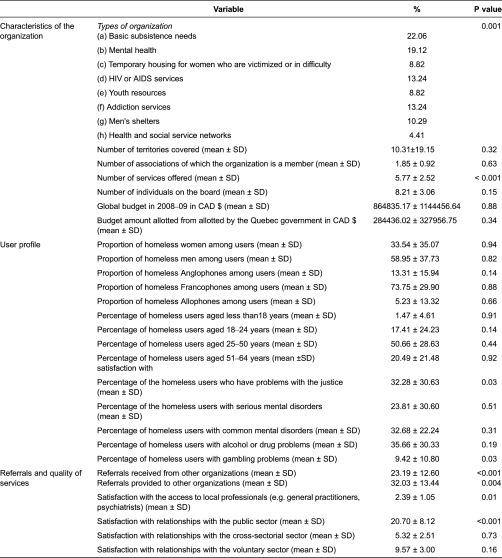
Characteristics of the sample and their association with interorganizational collaboration (*n*=68)

Abbreviations: SD, standard deviation. HIV, human immunodeficiency virus; AIDS: acquired immunodeficiency syndrome.

**Table 2.**
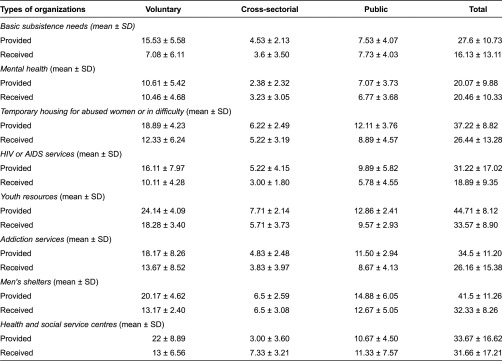
Mean referrals provided and received by types of organizations

**Table 3.**
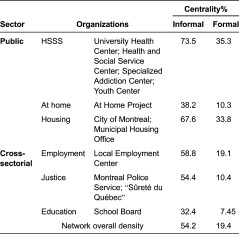
Statistical measurement of network centrality of collaboration between public and cross-sectorial organizations and the voluntary sector

Abbreviation: HSSS, health and social service sector

**Table 4.**
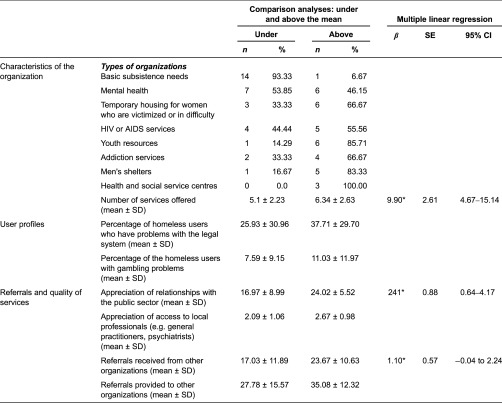
Variables predicting interorganizational collaboration

Note: Total variance explained: *R*^2^, 38.49%.*p<0.05.
